# The London Panel Committee

**Published:** 1914-10-03

**Authors:** 


### The London Panel Committee.

### POINTS OF POLICY DECIDED ON.

At a meeting of the London Panel Committee on Tuesday last, it was agreed to invite panel practitioners in the Metropolis to forward contributions not exceeding one guinea towards the cost of a motor ambulance to be presented to the British Red Cross Society. The Chairman, Dr. H. J. Cardale, in making the proposal, suggested that as the panel practitioners were receiving this week the £90,000 due in respect of persons who did not choose a doctor in 1913 it was fitting that a thankoffering should be made, and it could not take a better form at the present time.

The Committee considered the question of accepting membership of the Federation of Local Medical and Panel Committees proposed to be formed in connection with the British Medical Association. The draft constitution of the proposed Federation proposed that no single committee should take any steps of general importance to the Federation without previous consultation with the executive committee. On the executive London only had two representatives, and it was objected that as the Metropolis has special problems of its own it could not be restricted in its action in the manner proposed. The alliance of the Federation to the British Medical Association was also criticised, and it was felt that the calculation of the subscription on the basis of £1 for every 20,000 of the insured population would be unreasonably high in the case of London.

### Domiciliary Treatment.

The Insurance Commissioners having suggested that the Insurance Committee should adopt a scheme in connection with tuberculosis treatment under which the tuberculosis officer of a dispensary should be regarded as the consulting officer for the area, and practitioners on the panel should be required to submit forms and records to such officer, the Panel Service Sub-committee recommended that the President of the Local Government Board be asked to receive a deputation on this matter and the whole question of domiciliary treatment. A resolution was also passed reaffirming the opinion of the Panel Committee that panel practitioners should be represented on tuberculosis dispensaries to which grants of public money were made.

The Panel Service Sub-committee called attention to the fact that in Nottingham conferences between practitioners and representatives of approved societies had been the means of smoothing away many difficulties in connection with the administration of sickness benefit, and suggested that the Chairman of the Insurance Committee should be asked to summon a similar conference in London. The proposal was approved unanimously.

### Attitude to "Excessive Prescribing."

Considerable discussion took place on the vexed question of alleged excessive prescribing. As noted in reports in The Hospital before the summer recess, the Drugs and Appliances Sub-committee and the Pharmaceutical Committee have framed a formidable list of proprietary and other preparations which they wish to have excluded altogether from the scope of Insurance prescribing. The view taken by the Panel Committee, however, was that no non-medical body had a right to frame a list of drugs which doctors may not prescribe, and that the best way of dealing with the difficulty is to keep strict watch over the prescriptions and to require an individual practitioner to justify before his fellows any prescription which appears to be excessive either because it contains unnecessarily expensive drugs or is ordered in unduly large quantities. Resolutions expressing this opinion were passed and ordered to be forwarded to the Committees concerned.

### Non-panel Men and Representation.

Some time ago the Panel Committee decided to offer representation to members of the Local Medical Committee for London and to doctors representing the non-panel section of the profession. Almost unanimously the Committee accepted the Chairman's suggestion that in order to promote unity in the profession the Panel Committee should be the first to hold out the olive branch, and Dr. W. H. Burnhill, Mr. W. McAdam Eccles, and Dr. Walter Jagger were then elected.

